# Tracking the legacy of early industrial activity in sediments of Lake Zurich, Switzerland: using a novel multi-proxy approach to find the source of extensive metal contamination

**DOI:** 10.1007/s11356-022-21288-6

**Published:** 2022-06-29

**Authors:** Remo Luis Roethlin, Adrian Gilli, Bernhard Wehrli, Robin Sue Gilli, Jan Georg Wiederhold, Nathalie Dubois

**Affiliations:** 1 Department of Surface Waters Research and Management, Eawag, Überlandstrasse 133, 8600 Zurich, Dübendorf Switzerland; 2 Department of Earth Sciences, Geological Institute, ETH Zurich, Sonneggstrasse 5, 8092 Zurich, Zurich Switzerland; 3 Department of Environmental Systems Science, Institute of Biogeochemistry and Pollutant Dynamics, Aquatic Chemistry Group, ETH Zurich, Universitätstrasse 16, 8092 Zurich, Zurich Switzerland; 4 Department of Environmental Systems Science, Institute of Biogeochemistry and Pollutant Dynamics, Soil Chemistry Group, ETH Zurich, Universitätstrasse 16, 8092 Zurich, Zurich Switzerland; 5 Department of Environmental Geosciences, Centre for Microbiology and Environmental Systems Science, University of Vienna, 1090 Vienna, Vienna Austria

**Keywords:** Heavy metals, Lake sediment, Multi-proxy approach, Industrial pollution, Mercury, Stable mercury isotopes, Lake Zurich, Switzerland, Toxic metals, Trace metals

## Abstract

Historical industrial activities at the Horn Richterwil, on the shore of Lake Zurich (Switzerland), caused widespread metal contamination on land and in the adjacent lake sediments. This study provides an estimation of the age and source of the contamination by using XRF core scanning, ICP-OES, and Hg-AFS for quantitative measurements of trace metals and MC-ICP-MS for the stable isotope analysis of mercury. Radiometric dating ($^{137}$Cs, $^{210}$Pb, and Pu dating) of two proximal cores and varve chronology in a distal core suggest two different contaminations, one stemming from around 1960 (Zn, Cd) and an earlier one from 1880 (Cr, Cu, Hg, Pb, Sn). The XRF data suggest two different contamination pathways: one by landfill of contaminated soil and another one by industrial wastewater effluents. Maximum concentrations found within all samples are in the range of per mil (dry weight) for Cr, Cu, Hg, Pb, Sn, and Zn and lie within the top 10 cm of the sediment cores. The analysis of the mercury isotopic composition ($\delta ^{202}$Hg and $\Delta ^{199}$Hg) shows a significantly different signature for one of the cores, indicating a second mercury source. We could not identify the exact source or process leading to the isotopic fractionation of mercury, but the isotopic data confirm two different sources.

**Supplementary Information:**

The online version contains supplementary material available at 10.1007/s11356-022-21288-6.

## Introduction

Toxic metals in the environment are a global problem of rising concern due to their toxicity, their persistence, and their rising use globally (e.g. Thevenon et al. 2011). While potentially toxic trace metals are naturally occurring in the environment (e.g. geologic deposits, volcanic activity, or volatilisation from oceans), human activities have greatly accelerated their emission (Smol 2008). Early traces of anthropogenic influence on trace metal distribution were found, for instance, in Icelandic ice cores dating back to between 1100 BCE and 800 CE (McConnell et al. 2018). However, the most significant increase in metal contamination started with the industrial revolution (c. 1850). Important sources include the combustion of coal and other fossil fuels, the use of leaded gasoline (beginning in 1947 in Switzerland) (Christoph Moor 1996), the smelting of ores, and waste emissions of industries using metals in their processes, such as paper mills or chloralkali process plants. Pathways of metals emitted into the environment by industrial activities include atmospheric distribution, transport via water bodies, and direct deposition in landfills. Metals suspended in the atmosphere can reach surface waters, where they are scavenged by particles and transported to the sediments, as described by Shotyk et al. (1996) and Sigg et al. (1987). Particle-bound metals can become enriched in marine and lacustrine sediments, and biogeochemical cycling of trace metals also affects metal profiles in sediment records (Birch et al. 1996). Therefore vertical profiles of trace metals in sediments usually reflect variations in deposition over time unless they are affected by post-depositional processes. Chemical analyses and dating techniques allow using sediment records to reconstruct the contamination history and estimate the amount of potentially toxic metal deposited.

**Fig. 1:**
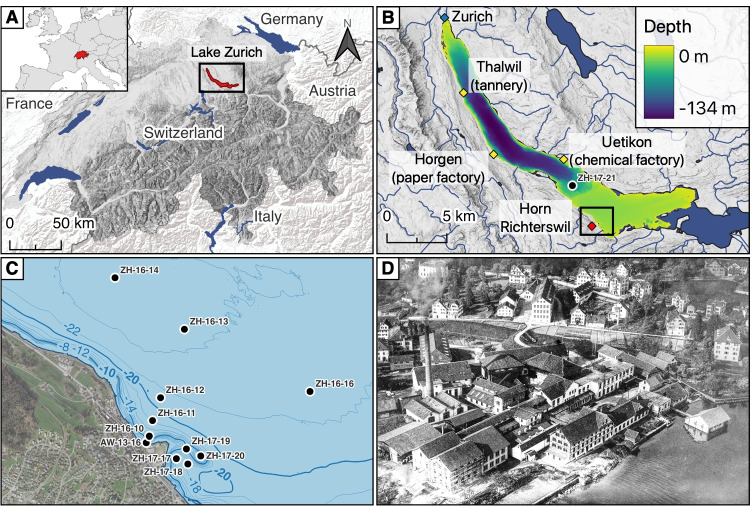
Location of Switzerland in Europe (inset) and Lake Zurich within Switzerland (**A**), close-up of Lake Zurich with bathymetry map and its surrounding surface water bodies. A small peninsula in Richterswil, called Horn (red diamond), is the focus of this study. Polluted sites are marked with a yellow diamond (**B**). Close-up of Horn with coring locations (**C**). Aerial photograph of Horn Richterswil showing factory buildings from est. 1918–1937 (**D**). World map: GADM Database v3.4 (www.gadm.org). Maps of Switzerland: Federal Office of Topography swisstopo. Photograph: ETH-Bibliothek Zurich, Bildarchiv/Stiftung Luftbild Schweiz / Fotograf: Mittelholzer, Walter / LBS_MH03-1597 / Public Domain Mark (http://doi.org/10.3932/ethz-a-000493539). Created with QGIS v3.22 (QGIS Development Team 2016)

Switzerland, with its large lakes (Fig. 1), provides many lacustrine sediment records, serving as archives of anthropogenic influences in the past. Sediment cores of Lake Walen exhibited increased concentrations of lead and zinc throughout the lake, dating back to the beginning of industrialisation in Switzerland (Liechti 2015). Lavrieux et al. (2017) found increased levels of trace metals (Pb, Zn) in Lake Joux in the northwestern part of Switzerland and linked it to the advent of the watchmaking industry in the region. Tin contamination was found in several sediment cores of Lake Zurich, dating back to the late nineteenth century (Amsler 2009). During the establishment of the inventory of polluted sites for the canton of Zurich, different contaminations have been found near former industrial sites, leading to the discovery and investigation of industrial contaminations in Lake Zurich (Fig. 1). Several of these contaminated sites comprise metal contaminations, such as the ones in Horgen (paper factory), Uetikon (chemical plant producing sulphuric acid and later zeolites), Thalwil (tannery), and Richterswil (AWEL 2019).

The focus of this study is the contamination at Horn Richterswil, which has been back-filled with material to its current form in 1841. In 1854, a silk factory was erected on the Horn and active until 1862, after which different textile factories used the buildings to dye silk. In 1926, the factory building was sold to a cotton printing factory. The new factory was only active until 1929 when another company bought the lot and started producing rubber. This rubber factory existed at this place until 1976 but decreased production in the 1960s. The canton of Zurich purchased the lot on the Horn in the same year (1976), and it serves now as a recreational area for the public.

The Office of Waste, Water, Energy, and Air of the Canton of Zurich (AWEL) started investigating the site in 2013. It was found to be contaminated with potentially toxic trace metals and persistent organic pollutants. In 2015, contaminated soil on the Horn was dredged, and the public was informed about the legacy contamination. Moreover, surface sediment from a 50 m$^2$ area was withdrawn by suction from the shore around the Horn (AWEL 2016a). Analyses of the sediments carried out on behalf of the agency in 2016 indicate a broad distribution of several potentially toxic metals (Pb, Cd, Cr, Cu, Ni, Zn, As, Sn and Hg) around the Horn, with a maximum of contamination just at the shore (AWEL 2016, personal communication). This study aims to thoroughly assess the trace metal contamination in sediments around the Horn in Richterswil, including dating the contamination and assigning it to one or several sources.

## Methods

### Coring and sample preparation

Fourteen sediment cores were retrieved during two coring campaigns. Cores ZH-16-10–ZH-16-16 were recovered on 21 December 2016 and cores ZH-17-17–ZH-17-23 were taken on 7 April 2017 (coordinates in Appendix B Table 6). Core AW-13-16 was collected on 17 May 2013 for AWEL for a previous study. Core locations were chosen with regard to water depth, distance to the shore and the extent of the known contamination (Fig. 1). All cores were retrieved using a 63 mm diameter gravity corer. GPS coordinates were recorded with the ship’s navigational GPS, and depth was estimated with the ship’s echo sounder. The cores were opened longitudinally in the laboratory and stored at 4$^{\circ }$C. Core halves were photographed (line scanned) using a Jai CV L105 3CCD Colour Line Scan Camera in an Avaatech XRF core scanner. Photos were processed with Adobe Photoshop. Cores ZH-16-10, ZH-16-11, ZH-17-17 and AW-13-16 were sub-sampled at 0.5 cm intervals. The material was frozen for 24 h, freeze-dried for 48 h and finally ground using a mortar. For mercury-related analyses and elemental analyses, the powder was ground with a Retsch RS1 vibration disk mill (1400 rpm, 15 s) using a tungsten carbide disk to minimise inhomogeneity.

### Dating

Biogenic varves were counted in core ZH-17-21, retrieved from a deeper part of the lake (65 m water depth, location shown in Fig. 1). Several mm-thick Melosira (M. granulata) algae blooms which occurred in 1906 and 1982 in Lake Zurich were used as distinct event markers (Gammeter 1997; Nipkow 1927). Radiometric dating with $^{137}$Cs and unsupported $^{210}$Pb (Von Gunten and Moser 1993) was done for ZH-16-11 and AW-13-16 (15 and 24 samples measured, respectively) using a Canberra gamma spectrometer with high purity Ge well detectors at Eawag Dubendorf. Each sub-sample was measured for 24 h. Plutonium concentration ($^{239}$Pu + $^{240}$Pu) was measured for 18 sub-samples of AW-13-16 at Labor Spiez with ICP-MS following digestion.

### Non-destructive analyses

The Avaatech XRF core scanner was used to scan all core halves except for ZH-17-22 and ZH-17-23. It employs a rhodium (Rh) target for the X-ray tube, Ultralene 4 µm foil to cover the tube and detector system and is constantly flushed with helium. The energy dispersive detector (Canberra) can detect secondary radiation from elements Al to U (Richter et al. 2006). A certified standard was measured 10 times at the beginning of every measurement day. The down-core slit size was 5 mm, the cross-core slit size was 12 mm for all samples. Standard-resolution XRF scans were done with a step size of 5 mm and a count-time of 30 s, high-resolution XRF scans were done with a step size of 1 mm and a count-time of 90 s (only done for ZH-16-10, ZH-16-11 and ZH-17-17). The instrument parameters used to measure different elements are shown in Table 1. Mercury was added to the existing 30 kV method manually using the L$_{\alpha 1}$ line at 9.99 keV (Shibata et al. 2009).

**Table 1:** Parameters used for Avaatech XRF core scanner for different elements. * indicates artefacts caused by XRF tube and detector systems

Voltage [kV]	Current [uA]	Filter	Elements
10	1500	None	Al, Si, P, S, Cl, K, Ca
			Rh, Ti, V, Cr, Mn, Fe, Ba
30	2000	Pd thick	Ni, Cu, Zn, Ga*, Br, Rb, Sr,
			Y, Zr, Nb, Mo, Hg, Pb, Bi*
50	2000	Cu	Ag*, Cd, Sn*, Te*, Ba

Due to the inhomogeneity of the sample matrix (varying water content, bulk density, grain size), the counts received for each element are not proportional to counts of other elements or the counts of the same element in a different core (Hennekam and De Rick 2012; Tjallingii et al. 2007). XRF file handling and statistical analyses were done with GNU R v4.1.2 (R Core Team 2021) and the Tidyverse packages (Wickham et al. 2019).

### Geochemical analyses

Discrete samples from cores ZH-16-10 and ZH-16-11 were measured with a Spectro Arcos ICP-OES at Eawag for Cr, Cu, Pb, Sn, and Zn. A multi-element standard for Cr, Cu, Pb, Zn and a standard for Sn was used. The digestion of the powdered samples was achieved by adding 500 µL of 30% H$_{2}$O$_{2}$ slowly and 2 mL of 65% HNO$_{3}$ drop-wise to 100 mg sample in a Teflon tube. The samples were processed in an ultraCLAVE microwave reactor (MLS GmbH) at up to 250$^{\circ }$C and 110 bar for 2 h and diluted with nanopure water after the reaction had finished. Procedure error was estimated with replicates and found to be 10% or less in all cases but one (20%).

Total Hg concentrations were measured by atomic fluorescence spectrometry with cold-vapour introduction (Hg CV-AFS; Millenium Merlin System by PSA, Kent, UK). Two different procedures were conducted to extract mercury from the ground sub-samples: A total digestion with aqua regia (8 mL HCl 37%, 1 mL BrCl conc. stock solution (0.2 mol L$^{-1}$ BrCl in 37% HCl prepared after Bloom et al. (2003)) and 3 mL HNO$_{3}$ 69%) on one set of samples and a sequential extraction (Hall and Pelchat 2005; Wiederhold et al. 2015) with 6 mol L$^{-1}$ HNO$_{3}$ (first fraction, F1), and a subsequent digestion of the residue with aqua regia (second fraction, F2) on a second equivalent set of samples. F1 is thought to contain only non-HgS mercury (e.g. mercury bound to humic acids), while F2 also includes mercury from HgS. For the first step of the sequential extraction, 10 mL 6 mol L$^{-1}$ HNO$_{3}$ were added to 500 mg of the sample in a 50-mL PP centrifuge tube and left to react on a lateral shaker (Heidolph Multi Reax) for 2 h. Afterwards, the tube was centrifuged for 15 min at 3000 rpm. The supernatant was filtered through a 0.2-µm hydrophobic PTFE filter (conditioned with EtOH suprapur before and rinsed with nanopure water) and preserved by adding 1 vol-% conc. BrCl For the second step, 8 mL HCl 37% puriss. (drop-wise), 1 mL BrCl stock solution (0.2 mol L$^{-1}$ BrCl in 37% HCl puriss.) and 3 mL HNO$_{3}$ 69% suprapur was added to the residue. Ventilated parafilm patches were used to reduce evaporation and avoid spilling. The samples were then digested for 24 h at room temperature on a Heidolph Multi Reax shaker. Afterwards, 38 mL nanopure water was added to the mixture and centrifuged for 20 min at 3000 rpm. The supernatant was carefully removed and filtered through a hydrophilic 0.45-µm PTFE syringe filter. Dilutions were created in 1% BrCl solution (1% of stock solution in nanopure water) and aimed at a concentration of 1 ng mL$^{-1}$ (AFS calibration range: 0.1 ng mL$^{-1}$ to 5 ng mL$^{-1}$). BrCl stabilises Hg$^{2+}$ in the sample solution by oxidising elemental mercury and forming stable halogen complexes with Hg$^{2+}$. For the analysis, the Hg$^{2+}$ in solution was reduced to Hg$^0$ by mixing the sample solution with 2.5% (m/v) SnCl$_{2}$ (in 1 mol L$^{-1}$ HCl) and transported into the fluorescence spectrometer with an argon carrier gas stream. A washing solution of 1% BrCl (1% of stock solution in nanopure water) was used. The reference standard NIST Montana Soil 2711a was determined to be 7.29 ± 1.24 µg/g dry weight (n = 4). The certified concentration is 7.41 ± 0.18 µg/g. All given errors indicate $2\sigma$ of the measured replicates.

### Mercury isotope analysis

Mercury has seven stable isotopes ($^{196}$Hg, $^{198}$Hg, $^{199}$Hg, $^{200}$Hg, $^{201}$Hg, $^{202}$Hg and $^{204}$Hg), present in naturally occurring mercury (Wiederhold et al. 2015). Mercury exhibits both mass-dependent fractionation (MDF) and mass-independent fractionation (MIF) effects (Wiederhold 2015). While MDF effects can be observed in all isotopic systems, MIF effects are rarer and smaller. The nuclear volume effect is an example of MIF. It originates from non-linearity in size and shape differences of nuclei and arises from relativistic effects on the electronic structure of atoms (Wiederhold et al. 2010; Schauble 2007). Another example of MIF is the magnetic isotope effect which affects odd-mass isotopes with a nuclear spin and magnetic moment (Buchachenko 2001). It occurs mainly during radical-pair reactions and is linked to spin preservation during chemical reactions. Most prominently, it was found to be effective in photochemical reactions, which allows for two-dimensional mapping of isotopic fractionation (Hintelmann and Zheng 2011). For the MDF, the ratio of $^{202}$Hg to $^{198}$Hg is used ($\delta ^{202}$Hg). The delta zero reference material for this ratio is NIST SRM-3133. $\delta ^{202}$Hg can be calculated with the following equation (Wiederhold, 2015):

1 $$\begin{aligned} \delta ^{202}Hg = \left( \frac{\left( \frac{^{202}Hg}{^{198}Hg}\right) _{sample}}{\left( \frac{{^{202}Hg}}{{^{198}Hg}}\right) _{NIST~SRM-3133}}-1\right) \end{aligned}$$

Similarly, an isotope ratio can be calculated for different pairs of heavy and light mercury isotopes, usually with $^{198}$Hg as the light isotope. The MIF of $^{199}$Hg can be calculated by subtracting $\delta ^{202}$Hg from $\delta ^{199}$Hg using a theoretical mass-dependent scaling factor $\beta$ = 0.252. The Hg isotope MIF values for $^{200}$Hg, $^{201}$Hg and $^{204}$Hg are calculated accordingly with beta values of 0.5024, 0.752, and 1.493, respectively (Blum and Bergquist 2007). This difference $\Delta$ is then a measure for the MIF anomaly as calculated with the following equation:

2 $$\begin{aligned} \Delta ^{199}Hg = \delta ^{199}Hg - \beta * \delta ^{202}Hg \end{aligned}$$

Isotopic ratios were determined with a Plasma II MC-ICP-MS (Nu Instruments, Wrexham, UK) equipped with an HGX-200 cold-vapour introduction system (Teledyne CETAC, Omaha, USA) at the University of Vienna, Austria. The cold-vapour inlet works similar to the Hg CV-AFS introduction system, but a desolvating nebuliser (Aridus 2, Cetac) adds a Tl solution (NIST-997 with a certified isotopic composition) before injecting the stream into the plasma to correct for instrumental mass bias and instrument drift. The MC-ICP-MS is a magnetic sector mass spectrometer that can detect and measure elements with high ionisation potentials with multiple collectors, simultaneously measuring the different ion beams. Replicate measurements of an in-house standard (ETH Fluka Hg) relative to NIST 3133 were used to determine the accuracy and the long-term reproducibility over different analytical sessions (Wiederhold et al. 2015) (cf. Table S6 in SI of Brocza (2019)).

Measurement uncertainties represent $2\sigma$ of all measurements of the Fluka secondary standard from the analytical session in which the samples were measured (n = 12) and amount to 0.04 $\permille$ for $\Delta ^{199}$Hg and 0.29 $\permille$ for $\delta ^{202}$Hg respectively.

The total digestions and sequential extraction fractions (F1 and F2) of samples AW-13-16 (n = 1), ZH-16-10 (n = 3) and ZH-16-11 (n = 1) were measured for Hg isotope ratios.

### Elemental analyses

Total carbon was determined using a CHNS 932 automatic analyser (LECO, USA) in the soil chemistry group at ETH Zurich (measurement uncertainty calculated with sulphanilamide standard (n = 3) as $2\sigma = \pm 0.60 \%$). Inorganic carbon was measured using an SSM 5000A analyser (Shimadzu, Japan). Samples were put into a ceramic vessel and inserted into the analyser. The sediment samples were first exposed to hydrochloric acid and then heated up in a furnace (measurement uncertainty calculated with device standard (n = 4) as $2\sigma = \pm 0.53 \%$).

## Results

### Sedimentology

**Fig. 2:**
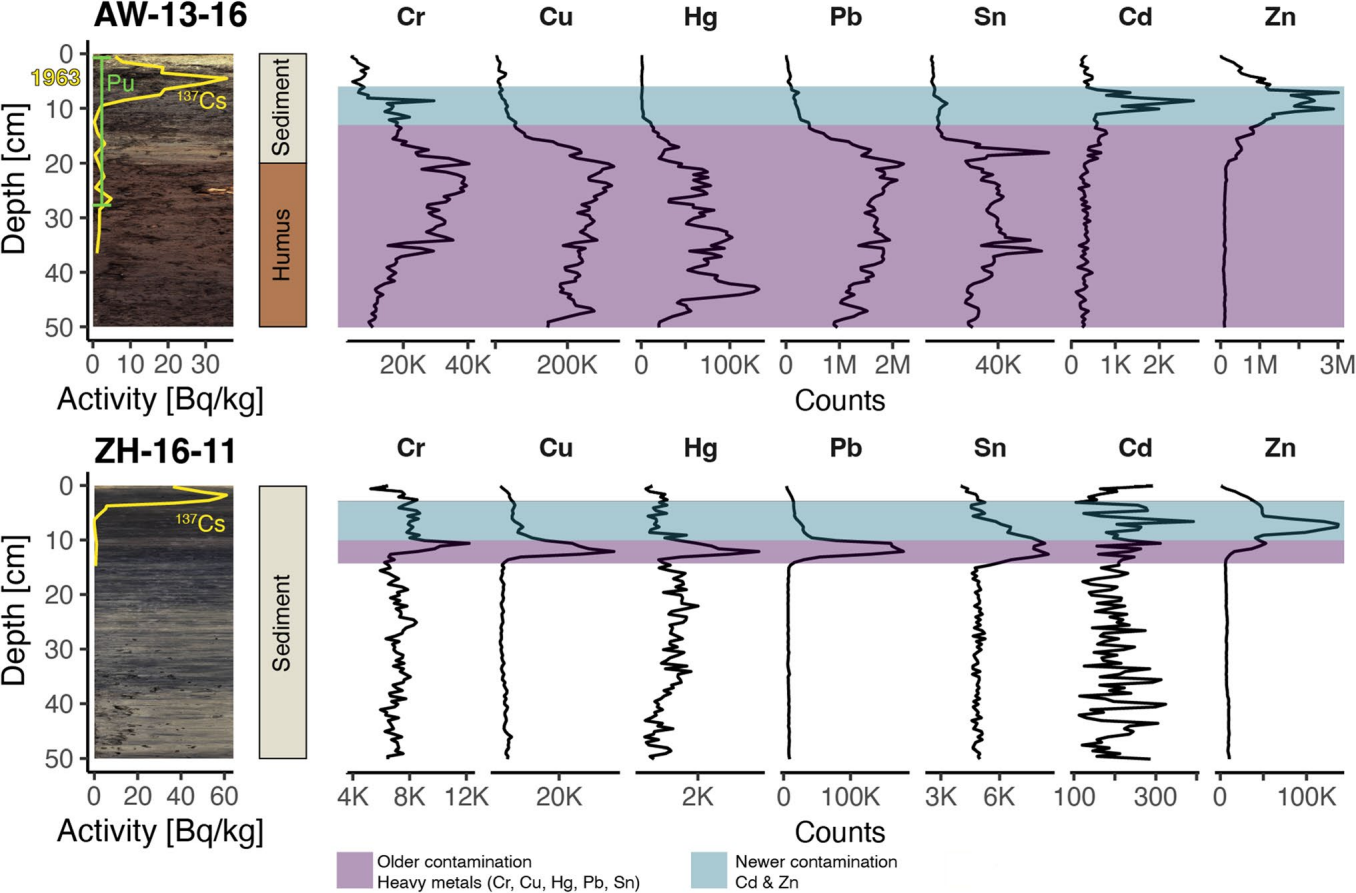
Overview of radiometric dating and relevant XRF traces for AW-13-16 and ZH-16-11. Shaded areas indicate correlating XRF signals (blue for Cd and Zn, purple for other metals)

The proximal sediment cores show remarkable stratigraphic differences, despite their low distance from each other. Figure 2 shows photographs of AW-13-16 and ZH-16-11 (close-up comparison in Appendix A Fig. 6). AW-13-16 contains finer layers of sediment on top (down to 20 cm), followed by humus (residues of a branch are visible). The humus has a distinct brown colour instead of the greyish clay top layer and did not react with hydrochloric acid. This layering suggests that the humus material was dumped into the lake and subsequently covered by lake sediments after deposition. ZH-16-11 presents a diffuse and disturbed lamination pattern with darker and brighter layers alternating and a very fine structure. The addition of a few drops of hydrochloric acid started a strong reaction suggesting a high carbonate content.

The comparison of all sediment cores (Appendix A Fig. 5) shows similar structures for cores ZH-16-10–ZH-16-16 and ZH-17-17–ZH-17-19; each shows a distinct change of colour from dark to bright layers (different aperture used in cores ZH-17-XX). However, an undisturbed and fine lamination is only visible for ZH-17-21–ZH-17-23.

### Dating

For AW-13-16 (Fig. 2), a maximum $^{137}$Cs activity can be found at 4.5 cm (35.6 ± 1.2 Bq/kg). A maximum Pu activity can be found at 6 cm (1.05 ± 0.08 Bq/kg) and no Pu activity could be measured below 27 cm depth. The $^{137}$Cs peak thus can be assumed to be from 1963 since the Chornobyl peak, usually found in Swiss lakes sediment, is not associated with a Pu peak (Appleby 2002). A mean sedimentation rate of 0.09 cm/year was obtained from unsupported $^{210}$Pb. However, since there is sediment missing towards the top of the core (no Chernobyl peak in $^{137}$Cs data), we refrain from using this average sedimentation rate and rely solely on the bomb peak (1963). The humus below 20 cm was most likely dumped before the start of atmospheric nuclear weapon testing in 1952 since the Pu signal in the humus is very weak.

For ZH-16-11, a maximum $^{137}$Cs activity of 61.1 ± 3.0 Bq/kg can be found at 1.75 cm. We assume this peak also corresponds to 1963. There is, however, most likely sediment missing at the core top, as we would expect to also see the Chornobyl peak (1986) in the $^{137}$Cs curve.

Sediment core ZH-17-21 was used to establish a varve chronology and to date the oldest contamination. The average sedimentation rate obtained through varve counting is 0.26 cm/year (Appendix A Fig. 7) and differs greatly from the sedimentation rates calculated from AW-13-16 and ZH-16-11. This can arise from the core location (assuming non-constant sedimentation rates throughout the lake), and disturbances in sedimentation or re-suspension in cores retrieved close to the shore.

### Non-destructive analyses

The highest counts of Cd, Cr, Cu, Hg, Pb, Sn, and Zn can be found in cores ZH-16-10 and AW-13-16 (Fig. 2), which are closest to the shore. Comparing the relevant XRF profiles of ZH-16-11 (and ZH-16-10) shows a remarkable correlation between Cd and Zn and Cr, Cu, Hg, Pb, and Sn. All contamination by trace metals is confined to the upper 15 cm of the core, with Zn and Cd showing two maxima at around 7 cm and 10 cm and the other metals having one peak at around 11 cm. The AW-13-16 XRF profiles show similar peaks in Cd and Zn in the top sediment part of AW-13-16 (6 cm to 11 cm), as is observed in the cores ZH-16-XX. There is a broad increase of Cr, Cu, Hg, Pb, and Sn from 15 cm onward with high maxima (Pb > 2 M counts). The broad increase corresponds well with the peculiar humus layer below 20 cm in AW-13-16. Visual inspection and correlation plots of XRF-scanned cores reveal a group of metals (Cr, Cu, Hg, Pb, Sn) and Zn and Cd to be correlating. Moreover, a large group of lighter elements often found in biogenic compounds, and terrestrial input elements correlate (Appendix A Fig. 8).

Appendix A Fig. 9 shows Pb and Zn traces for all cores. Shaded dots indicate possible equivalent peaks. The figure shows that ZH-16-10–ZH-16-16 present similar peak shapes at similar depths, whereas ZH-17-17 and ZH-17-18 share similar features. ZH-17-19 and ZH-17-20 show low counts and a Zn peak at the top of the core (ZH-17-19). AW-13-16 shares a similar-looking Zn peak but a different Pb characteristic. The peak shapes of Cr, Cu, Hg, Pb and Sn, as well as Cd and Zn, are very similar in core ZH-16-10 (Appendix A Fig. 8). The peaks of the same elements in ZH-16-11 are less pronounced but still match.

### Geochemical analyses

The concentrations measured with ICP-OES in the samples of ZH-16-10 and ZH-16-11 match well with the respective XRF curves (Appendix A Fig. 10). The maximum concentrations measured for different elements vary considerably: The highest Pb concentration found in ZH-16-10 is 10300 ± 188 µg/g dry weight, the highest Sn concentration in the same core is only 230 µg/g (Table 2).

**Table 2:** Measured maximum concentrations for ZH-16-10 and ZH-16-11 with ICP-OES (Cr, Cu, Pb, Sn, Zn). Error indicated by $2\sigma$. * means that no replicates were made

Sample ID	Concentration [µg/g]				
	Cr	Cu	Pb	Sn	Zn
ZH-16-10	1040 ± 21.1	1828*	10300 ± 188	230*	4632*
ZH-16-11	203*	241 ± 24.3	1190 ± 113	294*	822 ± 67.1

#### Hg-AFS

The peak Hg concentration measured in ZH-16-10 was determined to be 380 ± 110 µg/g dry weight, the maximum concentrations measured for ZH-16-11 and AW-13-16 were determined to be 1600 ± 60 µg/g and 3.08 ± 0.04 µg/g, respectively (no replicates).

**Fig. 3:**
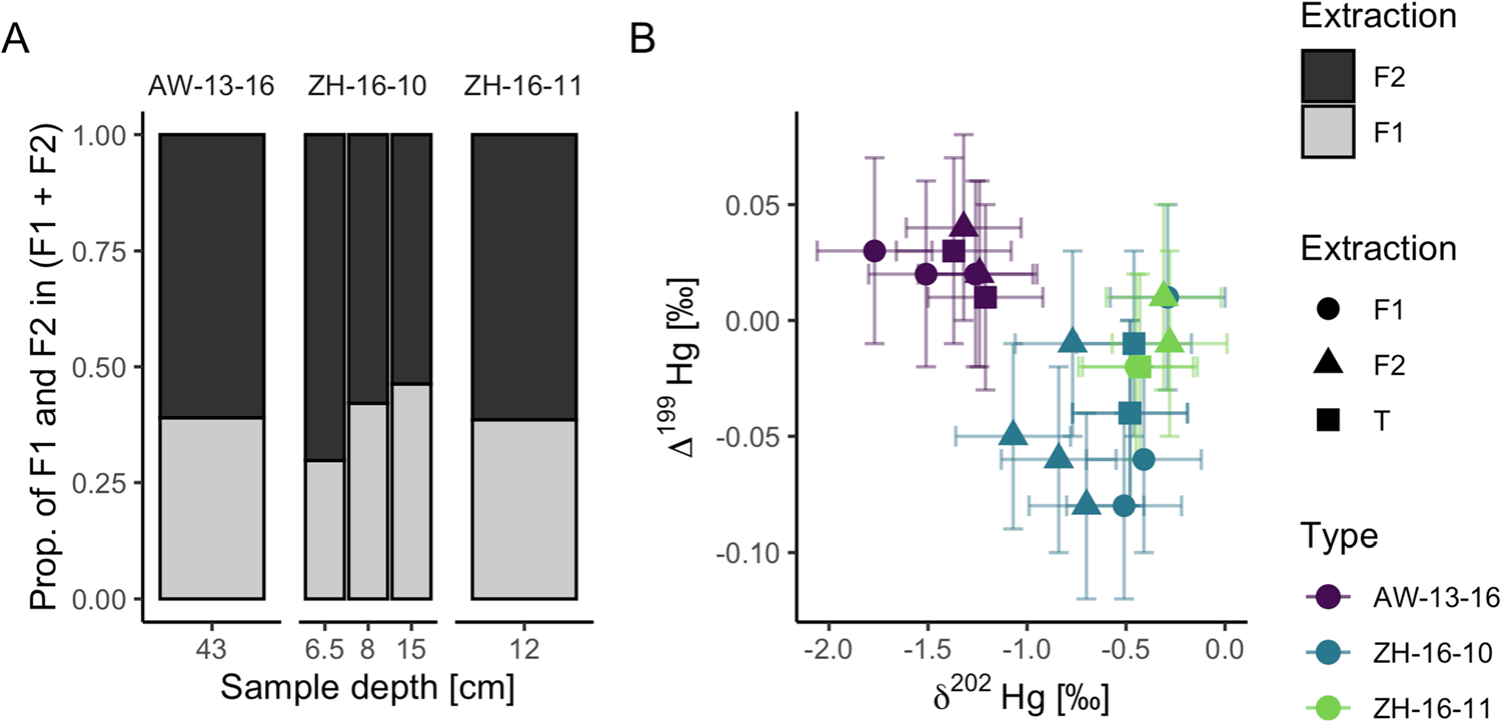
**A** Relative distribution of Hg pools for 6 mol/L HNO$_{3}$ extraction (F1) and aqua regia digestion of residue (F2) for sub-samples of AW-13-16, ZH-16-10 and ZH-16-11. Yield normalised to 1. **B**) Isotopic signatures of sub-samples of three different sediment cores. $\Delta ^{199}$Hg on y axis, $\delta ^{202}$Hg on x axis, referenced to NIST 3133 Hg isotope standard. Shapes with respect to extraction method. Measurement uncertainty indicated by crossbars is 0.04 $\permille$ for $\Delta ^{199}$Hg and 0.29 $\permille$ for $\delta ^{202}$Hg respectively. These values represent $2\sigma$ of all measurements of the Fluka secondary standard from the analytical session in which the samples were measured (n = 12)

The Hg concentrations measured in the fractions are illustrated as relative parts of 1 in Fig. 3 and tabulated in Table 3. The bar plot shows that the relative fractions are similar for every sub-sample, irrespective of core location or sample material (sediment vs humus). The discrepancy between Hg concentrations of F1 + F2 and T could arise from mercury loss during the extraction steps (e.g. syringe filters). The results suggest that the non-sulphidic to sulphidic mercury ratio is constant, regardless of the core location.

**Table 3:** Hg concentrations measured with Hg-AFS in total digestion (T) and sequential extracts of 6 sub-samples (F1, F2). * indicates that no replicates were made. Errors given as expanded uncertainty for 95% confidence with coverage factor k = 1.96. Relative extraction recovery normalised to total digestion

Core	Depth [cm]	Hg [µg/g]			
		T	F1	F2	Recovery (%)
AW-13-16	43-44	1600 ± 60	606 ± 8	950 ± 11	97%
ZH-16-10	6.5-7	125*	30 ± 2	76 ± 9	82%
ZH-16-10	8-8.5	380 ± 63	150 ± 15	205.26 ± 0.05	92%
ZH-16-10	15-15.5	3.6 ± 0.7	1.32 ± 0.02	1.4 ± 0.2	76%
ZH-16-11	12-12.5	3.08 ± 0.04	1.9 ± 0.2	1.17 ± 0.04	98%

The proportion of F1 and F2 for the two extraction steps (F1 and F2) is similar between sub-samples of one core and between cores (AW-13-16, ZH-16-10, ZH-16-11) (Fig. 3).

#### Correlation of XRF with ICP-OES and Hg-AFS

The XRF core scans from ZH-16-10 and ZH-16-11 were correlated with ICP-OES measurements (17 and 21 sub-samples, respectively) for Cr, Cu, Pb, Sn, and Zn. A correlation for Hg was only made for ZH-16-10 since only one Hg concentration measurement was available for ZH-16-11. For the correlations, 5 mm average values were calculated of the 1 mm XRF counts and then correlated with the ICP-OES measurements.

Pearson’s correlation coefficients between XRF and ICP-OES and Hg-AFS resp. are larger than *r* = 0.80 for ZH-16-10, except for Zn (Appendix B Table 7). Correlation coefficients are lower for ZH-16-11, with the Sn XRF not correlating with the ICP-OES measurements (*r* = 0.07). To predict concentrations of XRF counts, a simple linear regression model was used for ZH-16-10 and ZH-16-11 for all correlated elements. Appendix A Fig. 11 shows the regression model fitted to the data of ZH-16-10 with confidence intervals indicated (grey ribbons) and coefficient of determination R$^2$ shown. While there is a correlation between counts and concentration and most regressions are significant (at a 95% level), R$^2$ ranges from 0.360 (Zn) to 0.847 (Cr), and there seems to be a systematic error for all elements. This can be explained with a non-linear detection of elements by the XRF detector: The extensive range of measured counts makes it probable that some measurements lie outside of a linear range. Another explanation would be that the systematic error is caused by changes in water content, grain size and, most importantly, the sampling and aligning of these samples with the XRF curves. The simple linear regressions for ZH-16-11 (Appendix A Fig. 12) show similar coefficients of determination. There is no correlation for Sn, and the regression is not significant either.

The regressions need to be similar to predict concentrations with XRF counts for different cores and span an extensive range of XRF counts and measured concentrations. The regression plots show that most regressions are significant (F-test). However, the predicted concentrations vary significantly between ZH-16-11 and ZH-16-10. The regression coefficients for the linear equation are given in Appendix B Table 8.

#### Hg isotope analysis

The data in Fig. 3 shows that there are different isotopic fractionation patterns between sub-samples from different cores; however, only small differences are visible between the different extraction methods (Appendix B Table 9). A two-way ANOVA shows that there is a significant influence of the core type on the mass-dependent fractionation (MDF) $\delta ^{202}$Hg (p = < 0.001), but no influence of the extraction method on MDF. There is however an interaction effect between the sediment matrix and the extraction method, which is significant (p = < 0.01). The influence of the sediment matrix is also significant for the MIF (p = < 0.001), but not for the extraction method.

#### Elemental analysis

Total carbon and carbonate were measured for three samples in cores ZH-16-10, ZH-16-11 and AW-13-16, respectively. The comparison of total carbon and carbonate contents of the three sub-samples in Table 4 shows that the sample from AW-13-16 has a much higher total carbon content than ZH-16-10 and ZH-16-11. Moreover, AW-13-16 had a distinct odour similar to chlorinated organic solvents. These data suggest that the sub-sample from AW-13-16 is anthropogenically deposited soil likely contaminated with organic pollutants (odour, high organic carbon content). This finding matches well with the different morphological features presented in Fig. 2.

**Table 4:** Total carbon and carbonate analysis for three sub-samples. Total carbon measurements were done in duplicates, carbonate measurements were done in triplicates. Error indicates $2\sigma$

Core	Depth [cm]	Total carbon [% w/w]	Carbonate [% w/w]
ZH-16-10	20-20.5	9.7 ± 0.1	5.4 ± 0.4
ZH-16-11	0-0.5	12.00 ± 0.04	6.3 ± 0.6
AW-13-16	43-44	23.52 ± 0.07	0.4 ± 0.8

## Discussion

### Spreading of the contamination

**Fig. 4:**
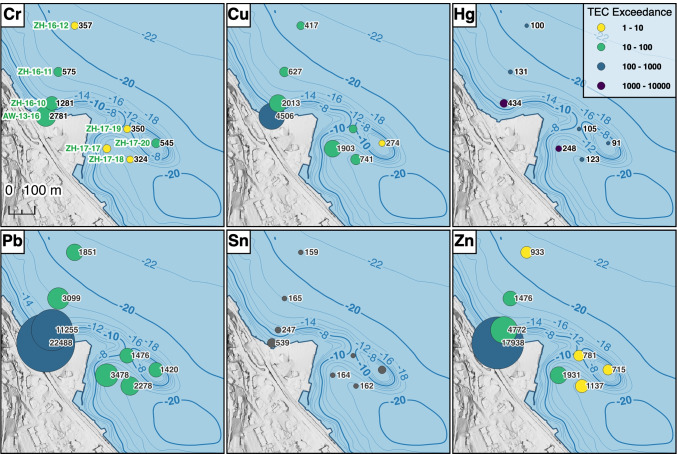
Distribution of metal contamination around Horn Richterswil. Colour of circles indicates times exceedance of respective consensus TEC (threshold effect concentration; no data available for Sn) value (cf. Table 5), size is proportional to estimated concentration in mg/kg (values noted next to circles). The core name is indicated in green together with a scale in the upper-left plot. Maps: Federal Office of Topography swisstopo. Created with QGIS v3.22 (QGIS Development Team 2016)

Using the XRF/ICP-OES/Hg-AFS data from ZH-16-10, concentrations can be estimated for all elements as shown in Fig. 4. It is evident that the majority of the contamination is located on the northwest side of the Horn, and only a lower amount of metals are found on the south-eastern side, where the contamination is also found at slightly deeper depths in the sediment column. As no cores from this location have been dated, a direct comparison is impossible. However, it may be that the upper sediment is not missing here, as is the case in the northern part (no Chernobyl $^{137}$Cs peak, see Section 4.2). The maximum contamination is, in any case, rapidly decreasing with increasing distance from the shore. The maximum concentrations of selected metals in ZH-16-11 are 5–10 times less than those in ZH-16-10, being 120 m away. A comparison of all XRF scans shows that there is still a signal of the contamination to be found in the distal core ZH-17-21, albeit much weaker (Appendix A Fig. 9).

### Age of the contamination

Sedimentation rates obtained by radiometric dating (AW-13-16 and ZH-16-11) and varve counting (ZH-17-21) vary considerably. This is expected since the distal core ZH-17-21 is located in a deeper, undisturbed part of the lake. The correlation of age models and XRF signals (Fig. 2 and Appendix A Fig. 9) shows that there are three different contaminations.

There is an older contamination including Cr, Cu, Hg, Pb and Sn dating back to c. 1880 (based on its appearance in core ZH-17-21, which has a varve chronology down to 1898) and a younger contamination (Cd and Zn) exhibiting two peaks from around 1950–1960 (Pu dating). The age of the first contamination was extrapolated in this core. The contaminations lie within a short period and are distributed similarly in sediment cores further away. This indicates that the contamination was introduced into the lake via industrial wastewaters.

The broad contamination (more than 40 cm) with no distinct concentration peaks in AW-13-16 of the humus layer below 20 cm depth indicates that contaminated material was dumped. It cannot be dated reliably using the results from radiometric dating. The Pu signal seen in the uppermost part of the dumped soil most likely migrated down from the overlying sediment, e.g. due to bioturbation.

### Source of the contamination

The metal contamination at Horn Richterswil most likely resulted from the industrial activities on the Horn. The most recent Zn contamination was most likely caused by the activities of the rubber factory (1929–1976). Zn is used as a catalyst and accelerator for the vulcanisation of rubber (Ghosh et al. 2003). The other metals found in the sediment cores, except those in the humus portion of AW-13-16, were probably caused by the textile factories. The printing, silk dyeing, and textile processing factories active on the Horn are known to have used potentially toxic metals and their salts as inorganic pigments and tanning agents during textile production (AWEL 2016, personal communication). Textile production is currently still using trace metals to this end (Liang et al. 2013). The highest concentrations of contamination found in any of the cores are located in the deposited soil of AW-13-16. One way to elucidate the contamination of this core, and relate it to the one found in the other cores, is to compare the mercury isotopic ratios with literature values. Previous studies have shown that the average global $\delta ^{202}$Hg composition for industrial Hg source materials varies considerably and is c. -0.65 ± 0.56$\permille$ with respect to NIST-3133 (Grigg et al. 2018) and that the global atmospheric $\delta ^{202}$Hg of elemental mercury is 0.24 ± 0.24$\permille$ (Jiskra et al. 2015). Feng (2019) assessed the mixing of mercury from a chloralkali plant (+0.42$\permille$ $\delta ^{202}$Hg) and mercury from a natural background (-0.49$\permille$ $\delta ^{202}$Hg) and shows that the speciation and isotopic composition of mercury samples allow for tracing of industrial mercury contamination from contaminated sediments to biota. Wiederhold et al. (2015) showed that the range of $\delta ^{202}$Hg isotopic composition can vary considerably within contaminated sites (-2.1$\permille$ to 0.6$\permille$ in sites related to the Swedish paper mill industry) and compared to contaminations from similar Hg industry (-0.2$\permille$ to -0.5$\permille$ in sites related to the Swedish chloralkali industry). This shows that there is a wide range of $\delta ^{202}$Hg involved with different processes and sources. Other studies have found that sediments in North American lakes track anthropogenic changes in mercury cycling and saw an increase in the global $\delta ^{202}$Hg and $\Delta ^{199}$Hg coinciding with industrialisation (Lepak et al. 2020) and that atmospheric deposition of Hg from coal combustion in Australian lake sediments allow for tracking of Hg point sources (Schneider et al. 2021). Further discussion about the use of Hg isotope signatures as tracers for anthropogenic Hg inputs into sediments can be found in Kwon et al. (2020) and Lee et al. (2021).

The reported values for industrial activities are in the range of the ones measured for ZH-16-10 and ZH-16-11 (Fig. 3, Appendix B Table 9). The low $\delta ^{202}$Hg of the AW-13-16 sample (-1.3 ± 0.29 $\permille$) is significantly different from the values in ZH-16-10 and ZH-16-11, suggesting a different source or treatment of the mercury waste. The mercury found in AW-13-16 is confined to the humus layers, while the sediment on top is not contaminated with mercury. The source could be soil from a different industrial site or soil from the premises.

### Risk posed by the contamination

All quantitatively measured metal concentrations in AW-13-16, ZH-16-10 and ZH-16-11 exceed the background concentrations estimated from the top 30 cm of sediment in Lake Zurich by AWEL (2016b). To further assess the risk posed by the different metals present in this contamination, we calculated how many times the estimated maximum concentrations exceed the respective consensus threshold effect concentrations (TEC) and probable effect concentrations (PEC), as proposed by MacDonald et al. (2000) (see colours of the circles in Fig. 4). Despite Pb exhibiting the highest concentrations around the horn, the pollution with the highest risk is Hg, which exceeds the respective TEC (cf. Table 5) in all displayed cores more than 100 times and up to 10000 times. Interestingly, the Lake Zurich background concentrations of the metals under investigation here are above the consensus TEC values and close to (for Cu, Hg and Zn) or above (for Cr and Pb) the PEC values.

**Table 5:** Maximum estimated contamination (in mg/kg), based on XRF/ICP-OES/Hg-AFS correlation of ZH-16-10. TEC/PEC (threshold effect concentration/probable effect concentration) values are indicated where available. Values in brackets represent maximum concentrations measured with ICP-OES/Hg-AFS

Core	Cr	Cu	Hg	Pb	Sn	Zn
AW-13-16	2781	4506	1891	22488	539	17938
			(1599)			
ZH-16-10	1281	2013	434	11255	247	4772
	(1042)	(1828)	(354)	(10311)	(230)	(4517)
ZH-16-11	575	627	131	3099	165	1476
	(169)	(241)	(3)	(1188)	(294)	(822)
ZH-16-12	357	417	100	1851	159	933
ZH-16-13	303	417	94	1620	163	839
ZH-16-14	354	375	99	1591	162	868
ZH-16-15	291	318	94	1605	179	862
ZH-16-16	330	315	95	1613	158	871
ZH-17-17	442	1903	248	3478	164	1931
ZH-17-18	324	741	123	2278	162	1137
ZH-17-19	350	415	105	1476	148	781
ZH-17-20	545	274	91	1420	-	715
ZH-17-21	417	440	103	1640	197	1050
Background	150	80	1	200	200	300
TEC	43.4	31.6	0.18	35.8	-	121
PEC	111	149	1.06	128	-	459

### Remobilisation potential

An assessment of the remobilisation potential of potentially toxic metals at this contaminated site needs to include different factors, such as bottom lake oxygenation, the strength of the adsorption of metals in sediments, water depth, depth of the contamination within the sediment, and probability of bioturbation.

Lake Zurich is a mesotrophic lake, and the oxygen concentration has been around 4 mg/L since 1994 (measuring point at 25 m below the surface near Stäfa, ZH). We have not observed any varves in sediment cores from shallow waters which leads us to expect some level of bio-activity and bioturbation around the Horn (Fig. 1, Fig. 4). The deeper part of the lake is still anoxic, as can be seen in the varved sediment core ZH-17-21 (Appendix A Fig. 7) (Gammeter 1997).

Metals such as Pb, Sn, Hg, and Cd are known to precipitate as heavily soluble sulphides in anoxic sediments (Christoph Moor 1996). In the case of mercury, this could be shown with the sequential extraction: F1 (extract with 6 mol/L HNO$_{3}$) should only contain non-HgS bound mercury, while F2 should consist of HgS bound mercury. The ratio between both fractions was stable (Fig. 3), indicating that the core location is not essential for the speciation of mercury in the sediment. Interestingly, the Hg in the dumped soil of AW-13-16 showed a similar ratio. The sequential extraction shows that the mercury in all samples is either bound to HgS or non-sulfidic Hg, presumably mostly NOM-bound Hg. This conforms with other research, which indicates that mercury is tightly bound to reduced sulphur and humic acids in soils (Charnock 2003; Skyllberg et al. 2006).

An important point is the depth of the contaminated sediment underwater and the depth of the contamination within the sediment. In this case, the contaminated sediments are located in shallow waters (20 m underwater), which increases the risk of remobilisation. While the contaminations within the sediments (Fig. 4) have been already covered with a layer of non-contaminated sediment (5 cm to 10 cm of sediment material), the upper part of the sediment is missing (no Chernobyl peak in $^{137}$Cs signal) at least in core ZH-16-11 and AW-13-16, which points to a certain remobilisation over the last decades. As the Horn has been used as a leisure and swimming place ever since it was bought by the canton of Zurich, remobilisation becomes more probable. While a physical remobilisation due to swimmers is not expected, underwater currents and waves could cause such a reorganisation. Since the metals are strongly bound to sulphides and humic acids, the exposure risk created by such a mechanical remobilisation is still low. An approach for the assessment of the exposure risk has been developed by AWEL in the meantime. These new guidelines ought to improve the assessment of polluted sediments (AWEL 2016b, 2019).

Bioturbation is most likely happening in shallow parts of the lake (such as around Horn Richterswil) and contributes to the fact that no varves can be seen in those sediment cores (Hsü and McKenzie 1985). The impact depth of bioturbation has previously been estimated to 20 cm in sediments of Marano Lagoon, Italy (Piani et al. 2005). The ongoing sedimentation and the depth of the contaminations make bioturbation of these metals nowadays less probable (Fig. 4).

## Conclusion

The sediments around Horn Richterswil are strongly contaminated by several potentially toxic trace metals. The contamination is split into three different, distinct contaminations: a more recent one with Zn and Cd, an older one with Cr, Cu, Hg, Pb, Sn and one in soil dumped very close to the shore (also Cr, Cu, Hg, Pb, Sn). Radiometric dating of cores around the Horn and a varve chronology of a distal sediment core suggest that the contaminations stem from around 1960 (Zn and Cd) and 1880 (Cr, Cu, Hg, Pb, Sn). All the metals present exceed the consensus TEC and PEC values, with Hg displaying concentrations up to 10000 times the TEC value. XRF analyses suggest that the metal contaminations in the sediment were caused by industrial wastewater emitted into the lake. The XRF scans also reveal that the contamination decreases rapidly with increasing distance from the shore. The younger contamination (Cd and Zn) is believed to stem from a rubber factory (1929–1976), while the older contaminations were probably caused by textile factories. The exact source of the contaminated dumped soil remains unclear. Hg isotope values suggest a different source than that of the mercury found in the sediment, but a literature review did not lead to any further conclusions. Dating of the contamination in the deposited soil was not possible. The contamination is located in sediments in shallow water (around 20 m below surface), which means that bioturbation or mechanical perturbation could take place. While there is nothing to suggest bioturbation is happening, remobilisation by lake bottom currents or waves seems probable and missing material of sediment cores close to the Horn (missing Chernobyl peak) indicates that this could have been the case over the past decades. Metals in the sediment are most likely bound to sulphides or humic acids and are unlikely to become soluble again. Because of this, the exposure risk posed by trace metals in the sediments around Horn Richterswil is low. Despite the dominance of stable Hg forms in the sediments, suggesting a low Hg mobility and thus a lower risk for adverse effects on the lake ecosystem, it needs to be considered that Hg can be methylated by microbial processes under anoxic conditions in lake sediments (Hsu-Kim et al. 2013; Yu and Barkay 2022). Methyl-Hg is very toxic and bioaccumulates in aquatic food webs (Tang et al. 2020). Thus, removing Hg-contaminated sediment layers also helps minimise the risk of methyl-Hg formation and its entry into the food chain. Polluted surface sediments around Horn Richterswil have recently been removed as it is now used for recreational purposes (public swimming area) (AWEL 2019).

## Supplementary Information

Below is the link to the electronic supplementary material.

Supplementary file1 (PDF 25.5 MB)

## Data Availability

Data is available at https://doi.org/10.25678/0005Y5.

## Code availability

Code necessary to reproduce any calculations and most figures is available at https://github.com/blaidd4drwg/richterswil_horn_pollution_study.

## Supplementary information

This article is accompanied by electronically available supplementary information.
