# Supplementary material for: Tracking the legacy of early industrial activity in sediments of Lake Zurich, Switzerland: using a novel multi-proxy approach to find the source of extensive metal contamination
Source: Environ Sci Pollut Res Int. 2022 Jun 29;29(57):85789–801. doi: 10.1007/s11356-022-21288-6 (PMC9668972; doi:10.1007/s11356-022-21288-6)
Supplement: Supplementary file 1 — Supplementary file1 (PDF 25.5 MB) [file 11356_2022_21288_MOESM1_ESM.pdf]

Supplementary information

Tracking the legacy of early industrial activity in sediments of Lake Zurich, Switzerland: Using a novel multi-proxy approach to find the source of extensive metal contamination

Remo Luis Roethlin[1,2*], Adrian Gilli[2], Bernhard Wehrli[1,3], Robin Sue Gilli[5], Jan Georg Wiederhold[4] and Nathalie Dubois[1,2]

[1*]Department of Surface Waters Research and Management, Eawag, Überlandstrasse 133, Dübendorf, 8600, Zurich, Switzerland.

[2]Department of Earth Sciences, ETH Zurich, Sonneggstrasse 5, Zurich, 8092, Zurich, Switzerland.

[3]Department of Environmental Systems Science, ETH Zurich, Universitätstrasse 16, Zurich, 8092, Zurich, Switzerland.

[4]Department of Environmental Geosciences, Centre for Microbiology and Environmental Systems Science, University of Vienna, Vienna, 1090, Vienna, Austria.

[5]Soil Chemistry Group, Institute of Biogeochemistry and Pollutant Dynamics, Department of Environmental Systems Science, ETH Zurich, Universitätstrasse 16, Zurich, 8092, Zurich, Switzerland.

*Corresponding author(s). E-mail(s): remo.roethlin@eawag.ch;
Contributing authors: adrian.gilli@erdw.ethz.ch;
bernhard.wehrli@eawag.ch; robinsue.gilli@fhnw.ch;
jan.wiederhold@univie.ac.at; nathalie.dubois@eawag.ch;

Appendix A Figures

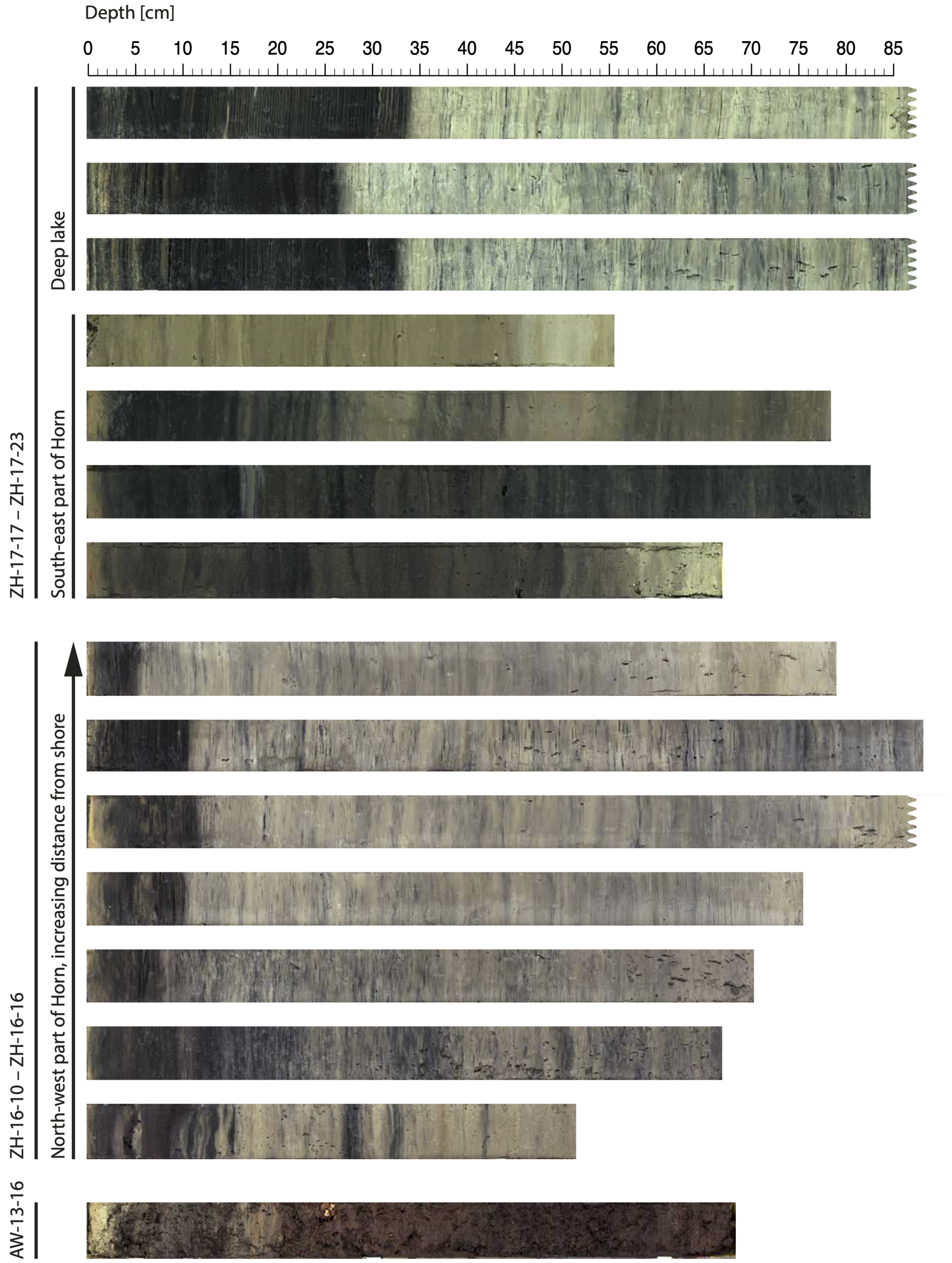

Fig. A1 Comparison of sediment cores AW-13-16, ZH-16-XX and ZH-17-XX. Zig-zag shapes at the bottom of the sediment core denote a cut off of the core.

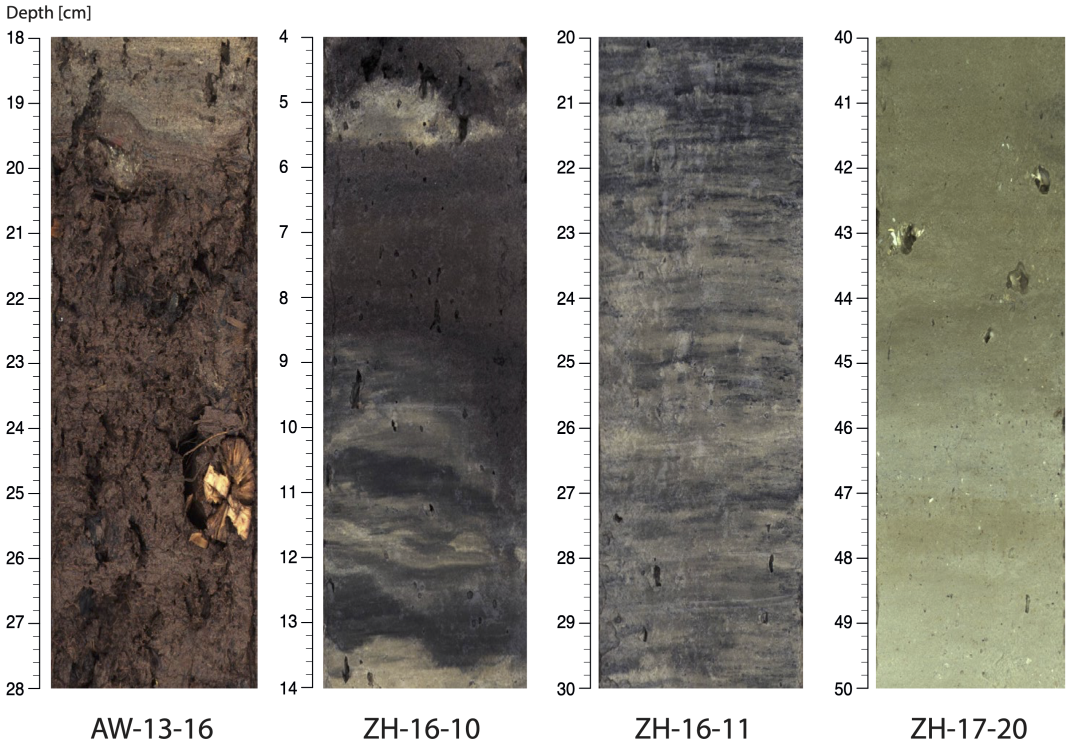

Fig. A2 Close-ups of sediment cores AW-13-16, ZH-16-10, ZH-16-11 and ZH-17-20.

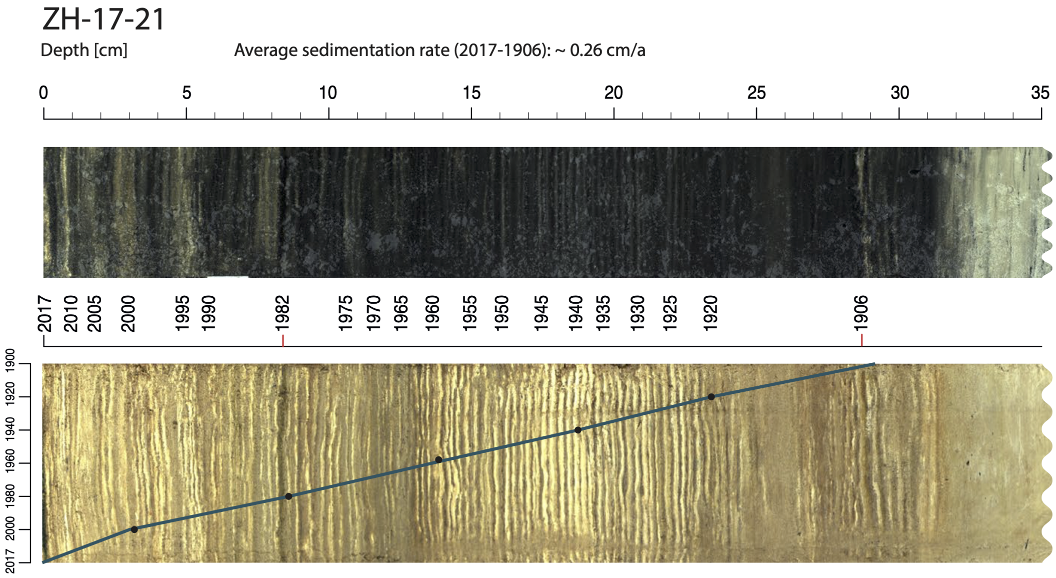

Fig. A3 Close-up of ZH-17-21 after opening (above) and after 48 h at air. Red lines indicate Melosira algae bloom events.

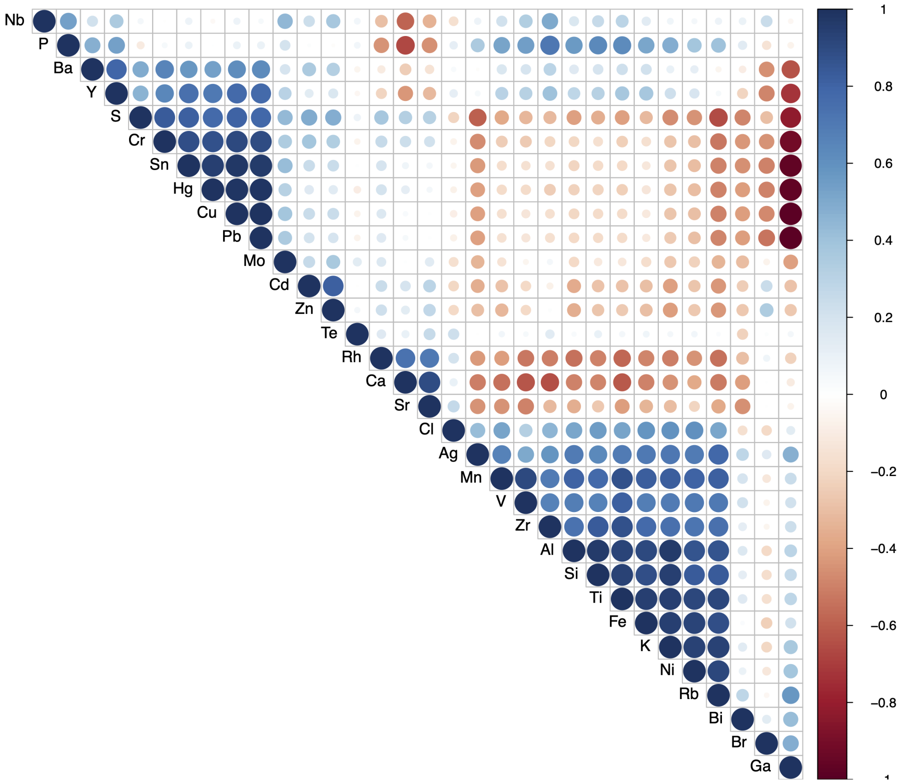

Fig. A4 Complete correlogram for XRF element traces in ZH-16-10. Size and transparency of dots are proportional to Pearson's correlation coefficient. Colour indicates syn- or anti-proportional correlation. Elements grouped by correlation.

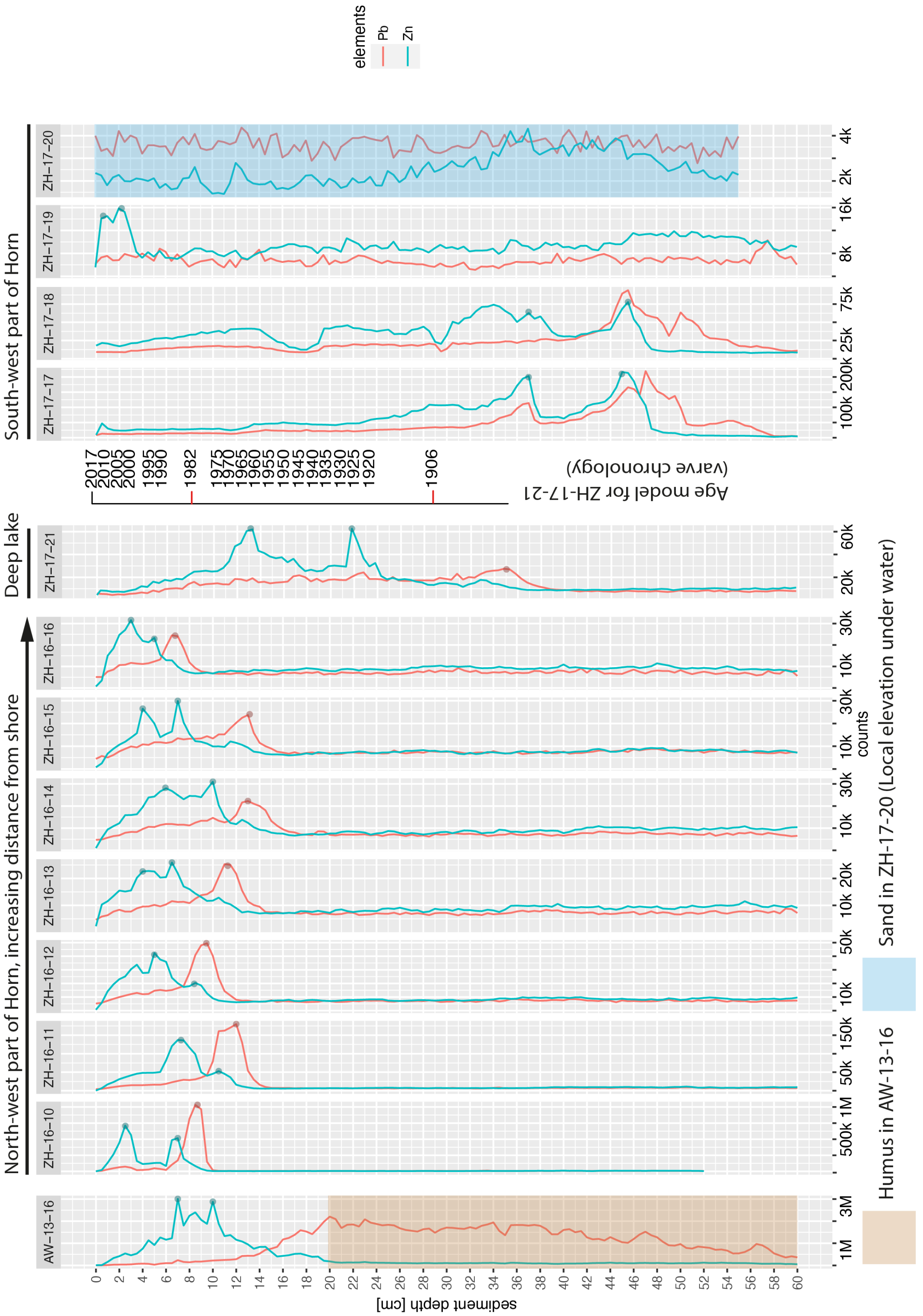

Fig. A5 Correlation of XRF traces of Zn and Pb for cores AW-13-16, ZH-16-10 – ZH-16-16 and ZH-17-17 – ZH-17-21.

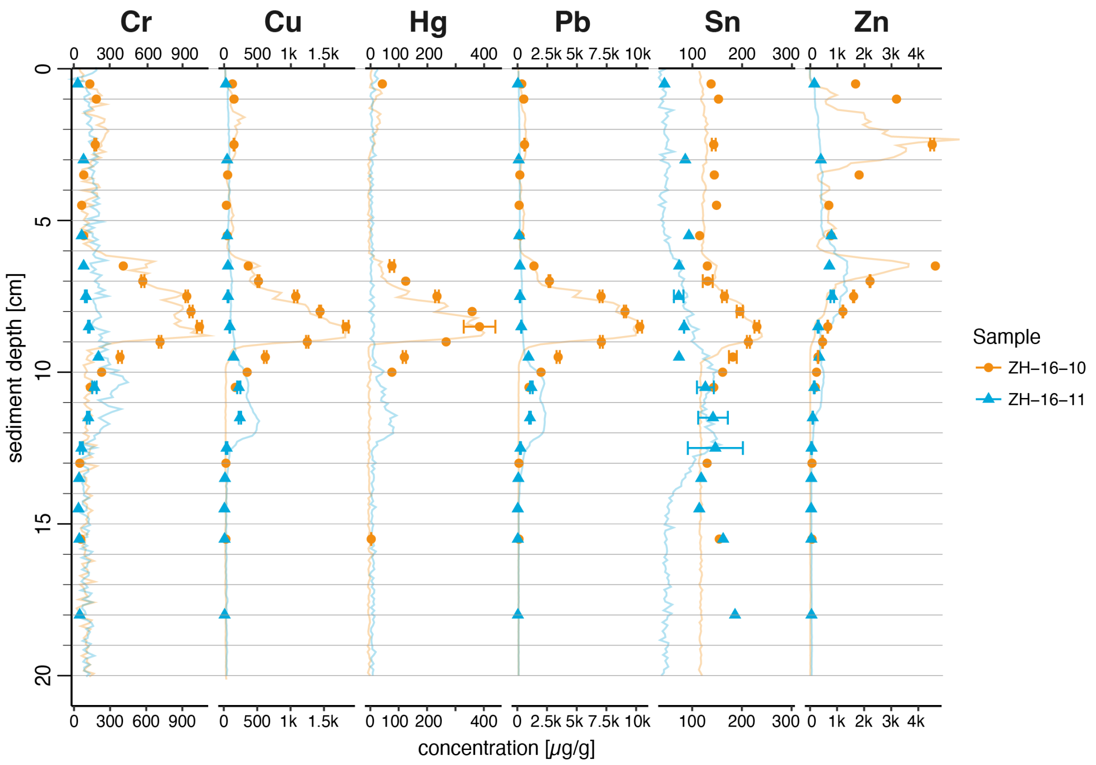

Fig. A6 ICP-OES measurements (Cr, Cu, Hg, Pb, Sn, Zn) for ZH-16-10 and ZH-16-11 and Hg-AFS measurements for ZH-16-10. Concentrations in µg/g dry weight. Error bars (2σ) are shown for duplicates (Hg-AFS) and triplicates (ICP-OES). XRF counts as shaded lines for comparison.

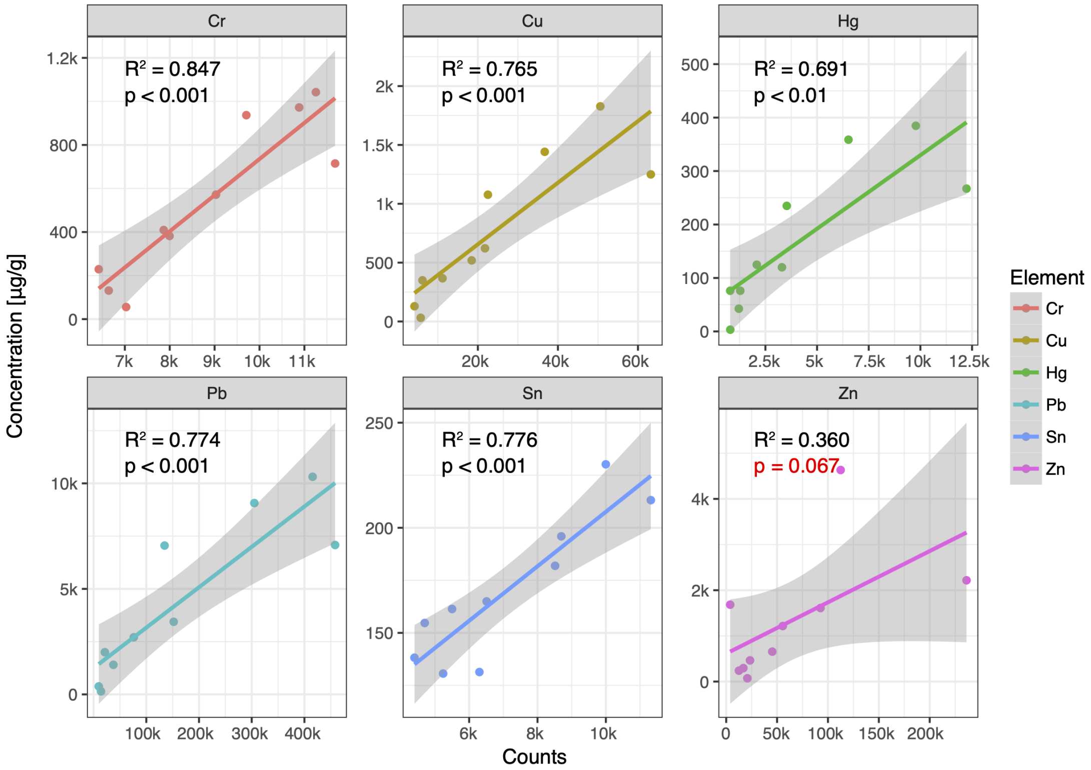

Fig. A7 Simple linear regression between XRF counts and measured concentrations (ICP-OES and Hg-AFS) for ZH-16-10. Grey ribbon indicates 95% confidence interval. R^2 denotes the coefficient of determination. P values indicate significance of the slope.

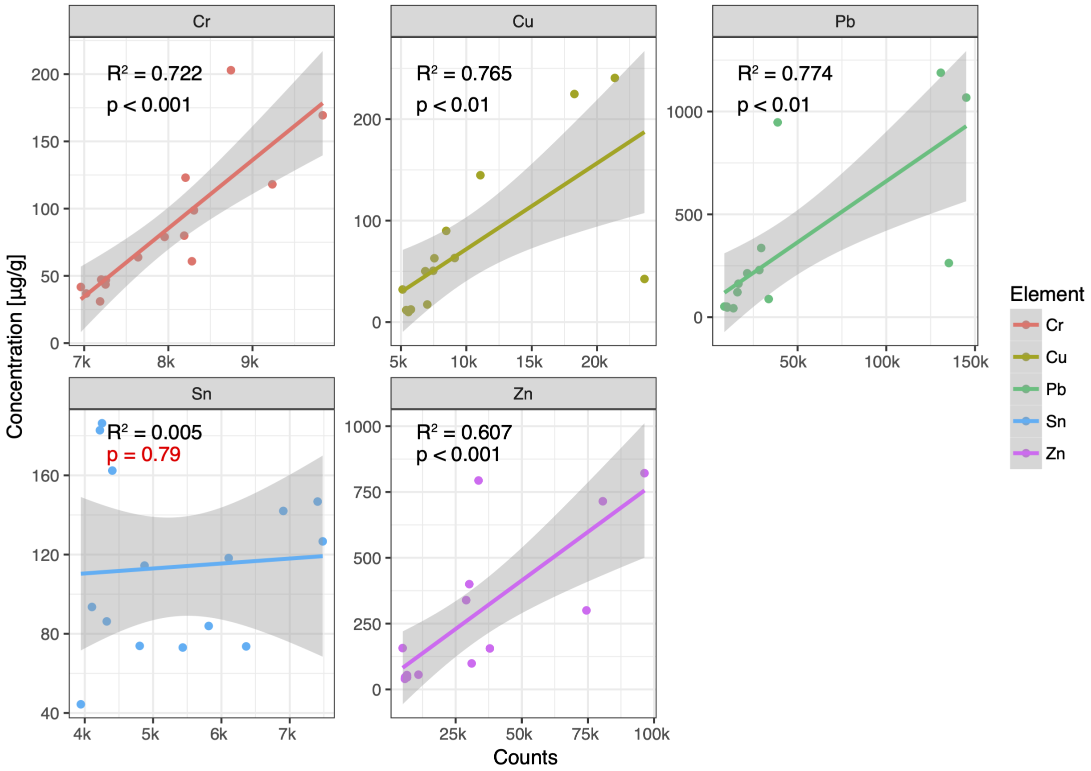

Fig. A8 Simple linear regression between XRF counts and measured concentrations (ICP-OES and Hg-AFS) for ZH-16-11. Grey ribbon indicates 95% confidence interval. R^2 denotes the coefficient of determination. P values indicate significance of the slope.

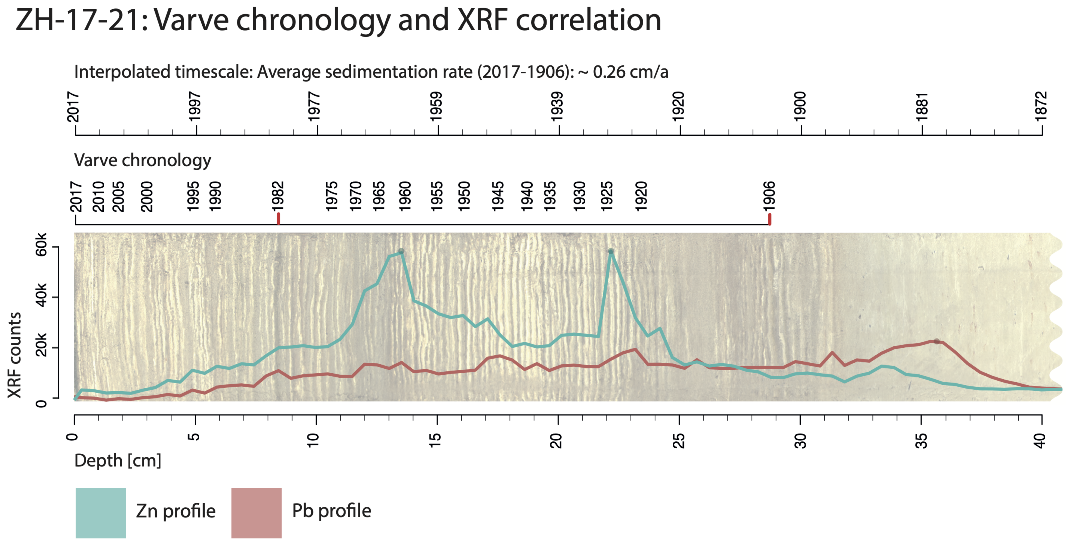

Fig. A9 Correlation of the ZH-17-21 varve chronology with Zn and Pb counts (XRF).

Appendix B Tables

Table B1 Sediment cores with corresponding CH1903 coordinates, core length and water depth. Depth recorded with ship's echosounder.

Core	Core length [cm]	Water depth [m]	CH1903 Y Coordinate	CH1903 X Coordinate
AW-13-16	67.5	n.a.	695883	229798
ZH-16-10	51.0	14.1	695907	229848
ZH-16-11	66.3	20.4	695932	229970
ZH-16-12	69.5	23.1	695995	230149
ZH-16-13	74.8	26.2	696182	230686
ZH-16-14	92.5	26.4	695638	231090
ZH-16-15	87.0	65.7	693997	233046
ZH-16-16	78.1	24.5	697167	230198
ZH-17-17	66.0	18.5	696119	229673
ZH-17-18	81.6	20.2	696209	229631
ZH-17-19	78.7	12.2	696198	229749
ZH-17-20	55.4	2.8	696310	229694
ZH-17-21	107.0	65.1	693887	232941
ZH-17-22	107.4	64.5	693704	233184
ZH-17-23	118.0	111	693140	234083

Table B2 Pearson's correlation coefficients for correlation of XRF scans with resp. ICP-OES measurements (Cr, Cu, Pb, Sn, Zn) and Hg-AFS measurements (Hg).

Element	Sediment core	
	ZH-16-10	ZH-16-11
Cr	0.92	0.85
Cu	0.87	0.69
Hg	0.83	-
Pb	0.88	0.74
Sn	0.88	0.07
Zn	0.60	0.78

Table B3 Regression coefficients for the regression between measured concentrations and XRF counts. Grey coloured values are not significant at a 95% significance level.

Element	ZH-16-10		ZH-16-11	
	α	β	α	β
Cr	-380 ± 147	$(7.83 \pm 1.18) \times 10^{-2}$	-321.0 ± 69.8	$(5.070 \pm 0.873) \times 10^{-3}$
Cu	$(1.93 \pm 1.48) \times 10^{2}$	$(1.310 \pm 0.256) \times 10^{-2}$	-12.7 ± 28.5	$(8.47 \pm 2.47) \times 10^{-3}$
Hg	66.7 ± 34.8	$(1.370 \pm 0.325) \times 10^{-2}$	-	-
Pb	$(0.138 \pm 8.220) \times 10^{2}$	$(9.57 \pm 1.83) \times 10^{-3}$	67.4 ± 96.8	$(5.94 \pm 1.50) \times 10^{-3}$
Sn	$(0.111 \pm 1.260) \times 10^{1}$	$(6.41 \pm 1.22) \times 10^{-3}$	100 ± 53	$(2.51 \pm 9.65) \times 10^{-3}$
Zn	$(6.90 \pm 4.69) \times 10^{2}$	$(5.74 \pm 2.70) \times 10^{-3}$	45.2 ± 69.7	$(7.37 \pm 1.64) \times 10^{-3}$

Table B4 Hg isotopic composition (in ‰) of measured samples. Run number indicates reruns of the same sample. Extraction type is indicated by T for total digestion and F1 and F2 for sequential extracts. Last line lists measurement uncertainties given as 2σ (n = 12) of the Fluka secondary standard from the analytical session in which the samples were measured.

CoreID	Name	Extraction	Run	δ^{202}Hg	Δ^{199}Hg	Δ^{200}Hg	Δ^{201}Hg	Δ^{204}Hg
AW-13-16	A16Hg1	F1	1	-1.77	0.03	0.09	0.07	0.02
AW-13-16	A16Hg1	F1	2	-1.51	0.02	0.13	0.08	-0.01
AW-13-16	A16Hg1	F1	3	-1.26	0.02	-0.09	0.01	0.02
AW-13-16	A16Hg1	F2	1	-1.32	0.04	0.08	-0.02	-0.01
AW-13-16	A16Hg1	F2	2	-1.24	0.02	-0.04	-0.03	-0.02
AW-13-16	A16Hg1	T	1	-1.37	0.03	0.10	0.03	-0.01
AW-13-16	A16Hg1	T	2	-1.21	0.01	0.02	-0.01	-0.01
ZH-16-10	Z10Hg10	F1	1	-0.51	-0.08	-0.04	-0.03	0.03
ZH-16-10	Z10Hg10	F2	1	-0.77	-0.01	0.10	-0.03	-0.03
ZH-16-10	Z10Hg10	T	1	-0.48	-0.04	-0.06	-0.05	-0.08
ZH-16-10	Z10Hg3	F1	1	-0.29	0.01	-0.13	-0.05	-0.05
ZH-16-10	Z10Hg3	F2	1	-0.84	-0.06	0.03	-0.04	0.01
ZH-16-10	Z10Hg3	F2	2	-1.07	-0.05	0.03	-0.02	0.00
ZH-16-10	Z10Hg3	T	1	-0.46	-0.01	-0.01	-0.03	-0.02
ZH-16-10	Z10Hg6	F1	1	-0.41	-0.06	0.00	-0.08	-0.01
ZH-16-10	Z10Hg6	F2	1	-0.70	-0.08	-0.17	-0.07	0.03
ZH-16-10	Z10Hg6	T	1	-0.48	-0.04	-0.01	-0.06	-0.03
ZH-16-11	Z11Hg5	F1	1	-0.45	-0.02	0.00	-0.03	0.04
ZH-16-11	Z11Hg5	F2	1	-0.31	0.01	0.14	-0.07	-0.07
ZH-16-11	Z11Hg5	F2	2	-0.28	-0.01	0.05	-0.09	0.00
ZH-16-11	Z11Hg5	F2	3	0.05	0.00	0.05	-0.05	0.02
ZH-16-11	Z11Hg5	T	1	-0.43	-0.02	0.02	-0.03	0.02
Fluka 2σ				0.29	0.039	0.15	0.062	0.042
